# Photodynamic Therapy for X-ray-Induced Radiation-Resistant Cancer Cells

**DOI:** 10.3390/pharmaceutics15112536

**Published:** 2023-10-26

**Authors:** Hiromu Ito, Yoshimi Shoji, Megumi Ueno, Ken-ichiro Matsumoto, Ikuo Nakanishi

**Affiliations:** 1 Quantum RedOx Chemistry Team, Institute for Quantum Life Science (iQLS), Quantum Life and Medical Science Directorate (QLMS), National Institutes for Quantum Science and Technology (QST), Chiba 263-8555, Japan; shoji.yoshimi@qst.go.jp; 2 Quantitative RedOx Sensing Group, Department of Radiation Regulatory Science Research, Institute for Radiological Science (NIRS), Quantum Life and Medical Science Directorate (QLMS), National Institutes for Quantum Science and Technology (QST), Chiba 263-8555, Japan; ueno.megumi@qst.go.jp (M.U.); matsumoto.kenichiro@qst.go.jp (K.-i.M.)

**Keywords:** radioresistance, reactive oxygen species, HCP1

## Abstract

Radiotherapy, in which X-rays are commonly used, is one of the most effective procedures for treating cancer. However, some cancer cells become resistant to radiation therapy, leading to poor prognosis. Therefore, a new therapeutic method is required to prevent cancer cells from acquiring radiation resistance. Photodynamic therapy (PDT) is a cancer treatment that uses photosensitizers, such as porphyrin compounds, and low-powered laser irradiation. We previously reported that reactive oxygen species (ROS) derived from mitochondria induce the expression of a porphyrin transporter (HCP1) and that laser irradiation enhances the cytotoxic effect. In addition, X-ray irradiation induces the production of mitochondrial ROS. Therefore, radioresistant cancer cells established with continuous X-ray irradiation would also overexpress ROS, and photodynamic therapy could be an effective therapeutic method. In this study, we established radioresistant cancer cells and examined the therapeutic effects and mechanisms with photodynamic therapy. We confirmed that X-ray-resistant cells showed overgeneration of mitochondrial ROS and elevated expression of HCP1, which led to the active accumulation of porphyrin and an increase in cytotoxicity with laser irradiation. Thus, photodynamic therapy is a promising treatment for X-ray-resistant cancers.

## 1. Introduction

In recent years, the number of patients with cancer has increased despite the development of novel materials and devices for medical care and diagnosis. Surgery, radiation therapy, or a combination of these therapies are often considered in cancer treatment. Although surgery is usually the first-line treatment in solid tumors, it may be difficult to perform due to various issues. The number of patients with cardiovascular diseases and brain stroke has been increasing with ageing populations, followed by an increase in the number of patients taking thrombolytics [1,2]. Given the risk of intraoperative bleeding, patients on antithrombotic drugs should reconsider undergoing surgery [3,4]. In addition, anticancer drugs are widely used as the main treatment modality in cancer therapy for shrinking the cancer tissue in patients unfit for surgery. However, anticancer drugs often affect the normal tissues, thereby reducing patient quality of life. Drug delivery systems have been developed to enhance the delivery of a therapeutic agent to its target site and reduce its side effects [5,6]. However, only a few examples of clinical applications are known, and the side effects have not been completely overcome. In addition, radiation can reduce the quality of life because of side effects such as skin injury and oral mucositis [7,8]. Recently, particle therapies and computer-assisted irradiation technologies have been developed and utilized in radiation therapy to reduce such side effects [9,10,11]. However, the cost-effectiveness of particle therapies is controversial, and it is difficult to eliminate these side effects completely [12,13]. Furthermore, radiation therapies require repeat applications and in some cases, cancer cells acquire radiation resistance due to irradiation [14]. Radiation resistance may induce cancer recurrence and is a poor prognostic factor [14,15]. Therefore, new therapeutic methods are required to prevent cancer cells from becoming radiation resistant.

Photodynamic therapy (PDT) is a cancer treatment that uses photosensitizers and low-power laser irradiation. Porphyrin compounds have often been used as photosensitizers for PDT because porphyrins specifically accumulate in cancer cells and reactive oxygen species (ROS) produced by energy transfer from the excited photosensitizer to molecular oxygen (O_2_) and exert anticancer effects [16,17]. Therefore, PDT is a relatively minimally invasive cancer therapy with no adverse effects. In addition, as no tissue incision is required, it is applicable in patients not suitable for surgery, such as elderly patients and those taking antithrombotic drugs. In addition, in neurosurgery, it is necessary to limit the resection area to prevent postoperative brain dysfunction. Photosensitizers are used in fluorescence-guided resection surgery [18]. However, the detailed mechanisms underlying porphyrin accumulation in cancer cells remain unclear. Heme carrier protein 1 (HCP1), characterized as a heme transporter, imports not only heme but also hematoporphyrins, and ROS derived from mitochondria are associated with the expression of HCP1 [19,20]. Thus, we suggest that the induction of ROS production in the mitochondria upregulates the expression of HCP1, leading to a subsequent increase in porphyrin accumulation, elevating the cytotoxic effect of laser irradiation in cancer cells. In addition, the generation of mitochondrial ROS is increased by X-ray irradiation of the cells [21]. Hence, X-ray-resistant cancer cells established by continuous X-ray irradiation may overexpress mitochondrial ROS and HCP1, such that PDT is effective in cancer therapy. Recently, PDT has been approved due to its effectiveness in the treatment of esophageal cancer, which is known to recur after radiation therapy; however, its mechanism is unclear. In this study, we used a rat gastric cancer cell line, RGK1, and X-ray-resistant RGK1 cells established by X-ray irradiation. The comparison of the therapeutic efficacy of PDT in terms of mitochondrial ROS production levels, expression of porphyrin transporters, and intracellular porphyrin accumulation between these cells conducted in this study provided valuable information on the possibility of noninvasive PDT on radioresistant cancer cells.

## 2. Materials and Methods

### 2.1. Materials

Dulbecco’s Modified Eagle Medium/Nutrient Mixture F-12, modified without l-glutamine, was purchased from Sigma-Aldrich (St. Louis, MO, USA). Fetal bovine serum was obtained from Cytiva (Marlborough, MA, USA). Penicillin/streptomycin was purchased from Thermo Fisher Scientific (Waltham, MA, USA). Hydroxyphenyl fluorescein (HPF) was purchased from Goryo Chemical, Inc. (Hokkaido, Japan). MitoSOX™ Mitochondrial Superoxide Indicators used as intracellular ROS detection were purchased from Thermo Fisher Scientific, Inc. To stain the mitochondria, MitoTracker Red CMXRos was purchased from Thermo Fisher Scientific, Inc. The following primary antibodies for Western blotting were commercially obtained: HCP1 antibody and β-actin antibody (Santa Cruz Biotechnology Inc., Dallas, TX, USA) and HIF-1α antibody (Cell Signaling Technology, Inc., Danvers, MA, USA). Horseradish peroxidase-linked anti-rabbit IgG and mouse IgGκ light chain binding protein conjugated horseradish peroxidase were purchased from Cell Signaling Technology, Inc. and Santa Cruz Biotechnology Inc., respectively, and used as a secondary antibody. For protein detection on the blotting membrane, the Immobilon Forte Western HRP substrate was obtained from Merck Millipore (Burlington, MA, USA). To obtain fluorescent images of cells, 35 mm glass-bottom dishes were purchased from MatTek Life Sciences (Ashland, MA, USA) and used in the microscopic experiments.

### 2.2. Cell Culture

The rat gastric cancer cell line RGK1 was established by Matsui et al. and kindly provided [22]. RGK1 cells were cultured in Dulbecco’s modified Eagle’s medium/nutrient mixture F-12 without L-glutamine (Sigma-Aldrich Co., LLC) containing 10% fetal bovine serum (Cytiva) and 1% penicillin/streptomycin (Thermo Fisher Scientific, Inc.). Cells were cultured in a humidified atmosphere under 5% CO_2_ at 37 °C.

### 2.3. Establishment of X-ray-Resistant Cells

X-ray-resistant RGK1 cells were established using multiple X-ray irradiations. RGK1 cells attached to 100 mm dishes were irradiated with X-rays using an MBR-1505R X-ray irradiation unit (Hitachi Medical Corporation, Tokyo, Japan). The tube voltage and current were 120 kV and 3.8 mA, respectively, and a 0.5 mm Al filter was used. The dose rate and radiation dose were 1 Gy/day and 2 Gy/day, respectively. Irradiation was performed 5 days/week for 30 days. After 30 d of irradiation, the surviving cells continued to proliferate and passed eight times. Resistance to X-rays was tested and compared to that of non-irradiated RGK1 cells using a colony formation assay. Briefly, the X-ray-irradiated cells and control non-irradiated cells were seeded on a 100 mm dish and incubated for 6 h. Each dish was irradiated with 0, 1, 2, 5, and 10 Gy X-rays at the rate of 1 Gy/min. The living cells were stained with a staining solution containing 2% (*w*/*v*) crystal violet, 0.8% (*w*/*v*) ammonium oxalate, and 20% (*v*/*v*) ethanol, and incubated for 5 min at 25 °C. The stained solution was then washed five times with Milli-Q water five times. The number of surviving cells was counted and viability was calculated.

### 2.4. Intracellular ROS Production

Intracellular ROS levels in X-ray-irradiated RGK1 cells were estimated and compared to those in control cells using hydroxyphenyl fluorescein (HPF) (Goryo Chemical Inc.). Cells were seeded on a 35 mm glass bottom dish at a density of $2\times 10^{5}$ cells/dish and incubated overnight. After incubation, the culture medium was replaced with a modified Hank’s balanced salt solution (HBSS), containing 10.0 mM HEPES, 1.0 mM MgCl_2_, 1.0 mM CaCl_2_, and 8.3 mM glucose, adjusted to pH 7.3. Then, cells were treated with 5 μM HPF and 200 nM MitoTracker^TM^ Red CMXRos (Thermo Fisher Scientific, Inc.) for 30 min at 37 °C. The fluorescence images of HPF and MitoTracker^TM^ were obtained by a CSU-10 confocal laser scanning unit (Yokogawa Electric Co., Tokyo, Japan) coupled to an Eclipse Ti-U inverted microscope with a PlanAPO 20× objective lens (Nikon Co., Tokyo, Japan) and a C5810-01 color chilled 3CCD camera (Hamamatsu Photonics K.K., Shizuoka, Japan) with excitation at 488 nm and 568 nm, and the emissions were filtered with 510–590 nm and 580–620 nm double-window barrier filters, respectively. The laser power and exposure time were set to 500 μW and 1.13 s, respectively. The fluorescence intensity of each cell was measured using IPLab Spectrum (version 3.0; Scanalytics Inc., Fairfax, VA, USA).

### 2.5. Detection of Mitochondrial ROS

ROS generation from mitochondria in the X-ray irradiated cells was detected and compared to the control cells by MitoSOX™ Mitochondrial Superoxide Indicators (Thermo Fisher Scientific, Inc.). The cells were seeded in a 96-well plate at a density of $1\times 10^{4}$ cells/well and incubated overnight. Cells were treated with 5 μM MitoSOX™ solution and incubated for 30 min at 37 °C. Fluorescence intensity was measured using an Infinite M200 microplate reader (Tecan Group Ltd., Männedorf, Switzerland) at 510 nm for excitation and 580 nm for emission.

### 2.6. Measurement of HCP Expression

The expression levels of HCP1, which functions as a porphyrin transporter, in X-ray-irradiated and control cells were compared by Western blot analysis. The cells were harvested and lysed with RIPA buffer containing 50 mM Tris-HCl (FUJIFILM Wako Pure Chemical Corporation, Osaka, Japan) solution (pH 8.0), 150 mM NaCl (FUJIFILM Wako Pure Chemical Corporation), 12 mM sodium deoxycholate (FUJIFILM Wako Pure Chemical Corporation), 3.5 mM sodium dodecyl sulfate ((FUJIFILM Wako Pure Chemical Corporation), 1% (*v*/*v*) Triton^®^ X-100 (NACALAI TESQUE, INC., Kyoto, Japan), and protease inhibitor cocktail (Takara Bio Inc., Shiga, Japan), and proteins were extracted on ice. The cell lysate was centrifuged at 12,000× *g* at 4 °C for 10 min, and the supernatant was used as a protein sample. The concentrations of proteins in the samples were adjusted between samples in advance using Pierce™ BCA Protein Assay Kits (Thermo Fisher Scientific, Inc.), according to the manufacturer’s protocol. A total of 15 μg cell lysate was mixed with Western blotting sample buffer containing 2-mercaptoethanol and heated at 95 °C for 5 min. The samples were applied to 10% (*w*/*v*) polyacrylamide gels, electrophoresed at 80 V, and transferred onto polyvinylidene difluoride (PVDF) membranes (Merck Millipore, Burlington, MA, USA). Membranes were blocked with 5% (*w*/*v*) skim milk dissolved in PBS containing 0.1% (*v*/*v*) Tween 20 (PBS-T). The membranes were exposed to reaction solution containing HCP1 antibody (Santa Cruz Biotechnology Inc.) diluted at 1:1000 and hypoxia-inducible factor 1α (HIF-1α) antibody (Cell Signaling Technology, Inc.) diluted at 1:1000 as primary antibodies and incubated at 4 °C overnight. The membranes were washed with PBS-T three times and further incubated with horseradish peroxidase (HRP)-linked secondary antibodies at 25 °C. The membranes were washed thrice with PBS-T. The blotting membranes were treated with Immobilon Forte Western HRP substrate (Merck Millipore) and chemical luminescence derived from the substrate and HRP was detected using FluorChemFC2 (Alpha Innotech Co., San Leandro, CA, USA). As a sample loading control, β-actin was detected. Blotting band intensities were calculated using an image processing software (ImageJ 1.52e).

### 2.7. Intracellular Porphyrin Accumulation Assay

The amount of porphyrin incorporated into the cells was compared between the X-ray irradiated and control cells. Cells were seeded in a 12-well plate at a density of $1\times 10^{5}$ cells/well and incubated overnight. The medium was replaced with a fresh one containing 20 μM hematoporphyrin dihydrochloride (HpD) and further incubated for 6 h. Cells were washed with growth medium twice and were lysed using RIPA buffer and the samples were moved to a 96-well plate. The fluorescence intensity of HpD was detected using an Infinite M200 microplate reader at 405 nm for excitation and 625 nm for emission.

### 2.8. Cytotoxic Assay

The effect of PDT on X-ray-irradiated cancer cells was also examined. X-ray irradiated and control cells were seeded in a 96-well plate at a density of $3\times 10^{3}$ cells/well and incubated overnight. The medium was replaced with a fresh one containing 20 µM HpD and further incubated for 6 h. After incubation, the cells were washed with growth medium and laser irradiation was performed using a 635 nm diode laser unit (HangZhou NaKu Technology Co., Ltd., Hangzhou, China) with an energy density of 2 J/cm^2^. The intensity of the irradiation power was measured using an optical multimeter (Ando Electric Co., Ltd., Kanagawa, Japan). After laser irradiation, cells were incubated for 24 h. After incubation, Cell Counting Kit-8 (DOJINDO LABORATORIES, Kumamoto, Japan) was added to each well. The absorbance at 450 nm was measured using an Infinite M200 microplate reader, and cell viability after PDT was determined.

### 2.9. Statistical Analysis

Statistical analyses were performed using the SPSS Statistics 28 software (International Business Machines Corporation, Armonk, NY, USA). Tukey’s HSD test was used to compare more than two datasets, and Student’s *t*-test was used to compare two datasets. *p* < 0.05 and *p* < 0.01 were considered as statistically significant differences. All data are presented as mean ± standard deviation.

## 3. Results

### 3.1. Confirmation of X-ray Resistance by Colony Formation Assay

**Figure 1:**
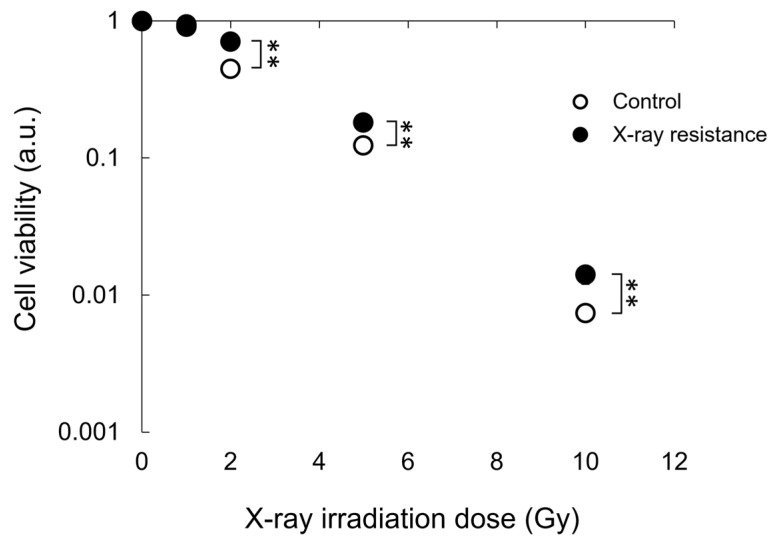
Cell viability after a single dose irradiation of X-ray was estimated and compared between the continual X-ray-irradiated RGK1 cells and the non-treated control cells by colony formation assay. Statistical significance was tested by Student’s *t*-test. *n* = 3, mean ± S.D., ** *p* < 0.01.

Resistance to X-rays in X-ray-irradiated RGK1 cells and control non-irradiated cells was confirmed by colony formation assay after X-ray irradiation. Figure 1 shows the cell viability calculated using a colony formation assay after X-ray irradiation. The cells repeatedly irradiated with X-rays in advance significantly suppressed the decrease in cell viability at 2, 5, and 10 Gy X-ray doses compared to the non-irradiated control cells. These results indicated that the X-ray-resistant RGK1 cell line could be successfully established.

### 3.2. Increase in Intracellular and Mitochondria-Specific ROS Production in X-ray-Resistant Cells

**Figure 2:**
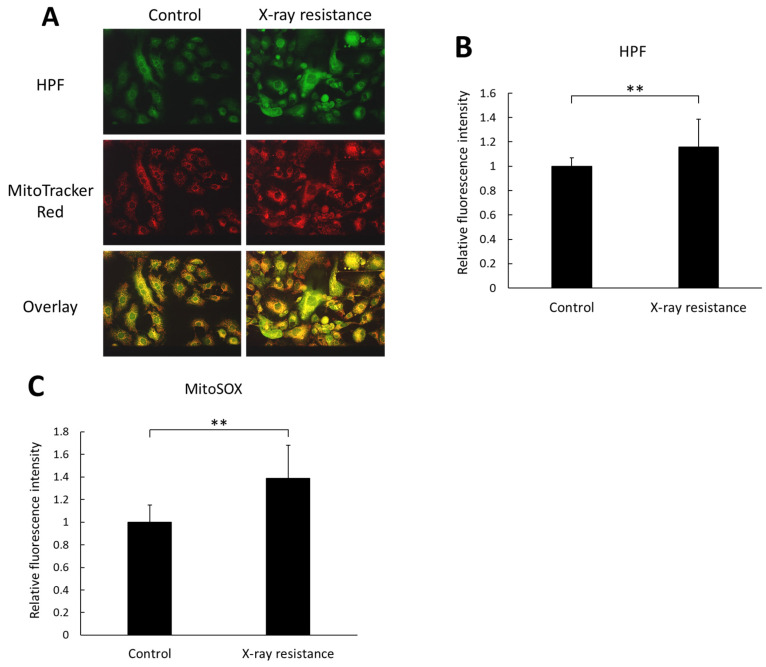
Intracellular ROS production level was estimated and compared between the X-ray resistant cells and the control cells. (**A**) representative images of cells stained with HPF and MitoTracker^TM^ Red CMXRos fluorescence dye. Scale bar: 50 μm. (**B**) the relative fluorescence intensity of HPF. (**C**) the relative fluorescence intensity of MitoSOX^TM^ Mitochondrial Superoxide Indicators. Statistical significance was tested by Student’s *t*-test. *n* = 120 for control samples in HPF experiment, *n* = 147 for X-ray resistant samples in HPF experiment, and *n* = 12 for MitoSOX^TM^ experiment. Mean ± S.D., ** *p* < 0.01.

Intracellular production of ROS in the X-ray-resistant cells and the control cells was detected by an HPF dye and MitoSOX™ Mitochondrial Superoxide Indicators, which indicate the generation of ROS derived from mitochondria. Figure 2A shows microscopic images of HPF and MitoTracker^TM^ Red CMXRos fluorescence in cells; HPF fluorescence coincided with the fluorescence of MitoTracker^TM^. Furthermore, the fluorescence intensity of HPF in X-ray-resistant cells was significantly higher than that in control cells, as shown in Figure 2B. These results indicate that the intracellular ROS level in X-ray-irradiated cells was greater than that in non-irradiated cells and that ROS production is likely from mitochondria because the localization of HPF green fluorescence in cells almost coincided with the red fluorescence of MitoTracker^TM^ Red CMXRos, which accumulates in mitochondria specifically by utilization of the mitochondrial membrane potential. Moreover, the HPF dye reacts with highly reactive oxygen species, such as hydroxyl radicals and peroxynitrite [23]. However, mitochondria are the major source of superoxide, and intracellular nitric oxide can react with superoxide to produce highly reactive peroxynitrite [24,25]. To investigate the location and species of ROS produced, a MitoSOX^TM^ assay was performed. MitoSOX^TM^ Mitochondrial Superoxide Indicators are highly selective for superoxide in the mitochondria, and their production in the mitochondria can be visualized using fluorescence microscopy. Thus, mitochondrial superoxide production in X-ray-resistant cells was detected and compared to that in non-irradiated control cells. According to the results of the MitoSOX^TM^ experiments, the fluorescence intensity of MitoSOX^TM^ increased in X-ray-irradiated cells, as shown in Figure 2C. Therefore, the generation of ROS increased in X-ray-resistant cancer cells, and the ROS produced in the cells originated mainly in the mitochondria.

### 3.3. Increase in HCP1 and HIF-1α Expressions in X-ray-Resistant Cells

**Figure 3:**
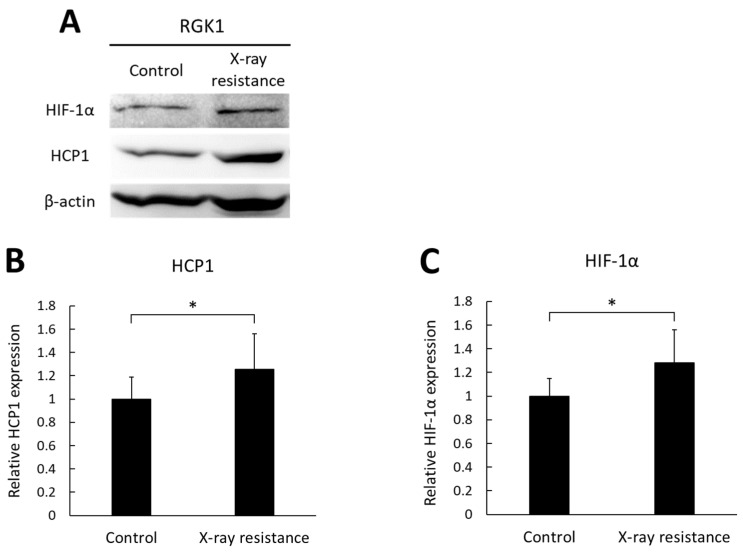
The expression levels of HIF-1α and HCP1 in the X-ray-resistant cells and control cells were analyzed by Western blotting. (**A**) representative images of blotting bands. (**B**) the relative expressions of HCP1. (**C**) the relative expressions of HIF-1α. Statistical significance was tested by Student’s *t*-test. *n* = 10, mean ± S.D., * *p* < 0.05. The uncropped blots are shown in Figure S1.

The expressions of a porphyrin transporter, HCP1, and a hypoxia-inducible factor, HIF-1α, were analyzed and compared between the X-ray-resistant cells and the non-irradiated control cells using Western blotting. HIF-1α is a subunit of HIF-1 protein and widely known as a marker of hypoxic condition. However, we have previously reported that HIF-1α could be an upstream factor of HCP1 [26]. Therefore, we confirmed the expression of HCP1 and HIF-1α in X-ray-resistant cells. Figure 3A shows representative images of blotting bands of HIF-1α, HCP1, and β-actin, and the relative expressions of HCP1 and HIF-1α were compared between the X-ray resistant RGK1 cells and control cells. Figure 3B,C shows relative mean intensities of blotting bands of HCP1 and HIF-1α. Both the expressions of HIF-1α and HCP1 were significantly elevated in the X-ray-resistant cells, compared to the non-irradiated cells. These results indicate that RGK1 cancer cells, which acquired X-ray resistance by continual irradiations of X-ray, induced increased expressions of both HIF-1α and HCP1.

### 3.4. Increase in Porphyrin Accumulation in X-ray-Resistant Cells

**Figure 4:**
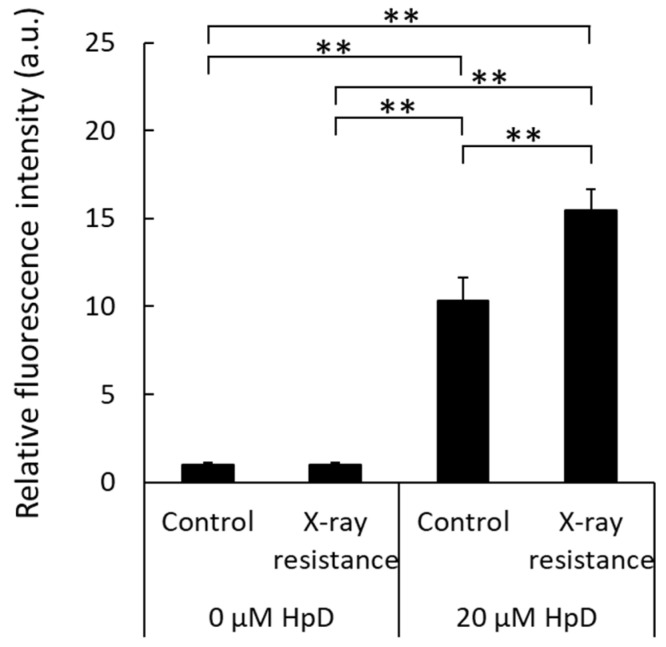
Comparison of intracellular porphyrin accumulation levels by measurements of porphyrin fluorescence in cell lysates. Statistical significance was tested by Tukey’s HSD test. *n* = 6, mean ± S.D., ** *p* < 0.01.

HCP1 is an import protein of hematoporphyrins, and its expression of HCP1 protein was increased in X-ray-resistant cells, as shown above. Therefore, intracellular porphyrin accumulation levels, which can be analyzed by measuring porphyrin fluorescence in cell lysates, were also elevated in X-ray-resistant cells. Figure 4 shows the relative fluorescence intensities. The samples without porphyrin treatment showed relatively lower intensities in both the X-ray-resistant cells and original control cells compared to the porphyrin-treated samples. Porphyrin treatment of the original cancer cells resulted in an increased intracellular fluorescence intensity. Moreover, a significant increase in fluorescence intensity was observed in X-ray-resistant cells compared to that in control cells. These results indicated that porphyrin accumulation levels in the X-ray-resistant cells were greater than those in the control cells.

### 3.5. Decrease in Cell Viability by PDT in X-ray-Resistant Cells

**Figure 5:**
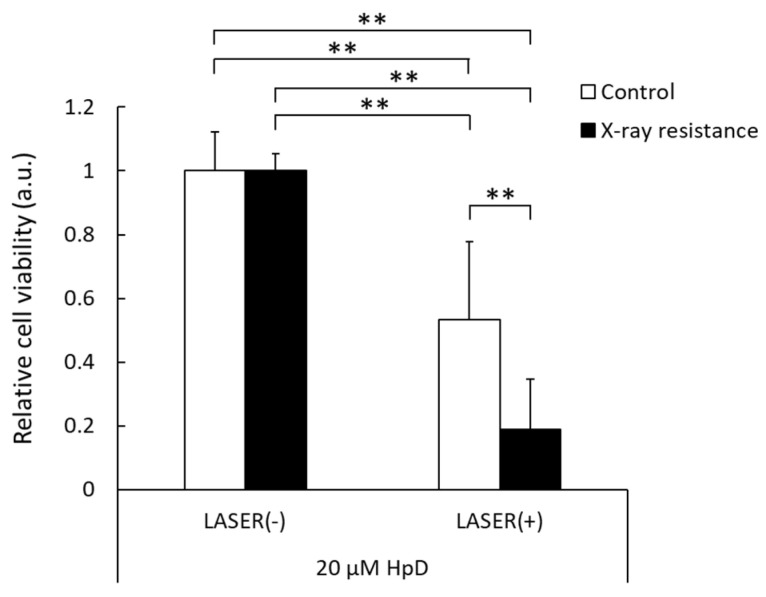
Relative cell viability with or without laser irradiation at 2 J/cm^2^ in the X-ray-resistant cells and the control cells. The viability is normalized with non-laser irradiated samples. Statistical significance was tested by Tukey’s HSD test. *n* = 6, mean ± S.D., ** *p* < 0.01.

The cell viability after PDT was analyzed, and the results are shown in Figure 5. In control RGK1 cells, the cell viability after laser irradiation decreased by approximately 50% compared to that of the samples without laser irradiation. However, the X-ray-resistant cells irradiated with laser showed much lower cell viability than cells without laser irradiation, and a significant difference was observed between the X-ray-resistant and control cells. These results indicate that PDT could be an effective treatment for X-ray-resistant cancer cells.

## 4. Discussion

Radiation therapy is a major method of cancer treatment, for which X-rays have been conventionally used. However, X-ray resistance during therapy can lead to tumor recurrence and a poor prognosis. In this study, we examined the effects of photodynamic therapy on X-ray-resistant cells induced by continuous X-ray irradiation. X-ray irradiation of cells generates ROS, which is related to the induction of apoptosis and cellular senescence [21,27]. Mitochondrial function is associated with apoptosis and senescence, and X-ray irradiation induces mitochondrial ROS production [21,25,28]. The X-ray-resistant cells used in this study were established with continuous X-ray irradiation, and an increase in intracellular ROS generation was confirmed (Figure 2A,B). In addition, the main source of intracellular ROS generation was the mitochondria because the intracellular localization of HPF fluorescence overlapped with that of MitoTracker^TM^ Red and an increase in MitoSOX^TM^ fluorescence was detected (Figure 2C). During irradiation, radiation might have damaged the mitochondria because ROS generation was elevated in resistant cells. However, the damage was limited because the membrane potential was normal based on the results of MitoTracker^TM^ (Figure 2A). Mitochondrial ROS have been reported to increase the expression of HCP1, followed by the elevation of porphyrin uptake in cells and cytotoxicity of PDT [20]. In contrast, HCP1 expression is enhanced in the murine duodenum under hypoxic conditions [29]. Hypoxia-inducible factor (HIF) is stabilized under hypoxic conditions. We previously reported that HIF-1α was an upstream factor of HCP1 and the stabilization of HIF-1α led to HCP1 expression [26]. It was confirmed that HIF-1α expression increased in addition to HCP1 expression in the X-ray resistant cells in this study as well (Figure 3). Additionally, mitochondrial ROS contribute to the stabilization of HIF-1α under hypoxic conditions [30]. Therefore, the HIF-1α stabilization induced by mitochondrial ROS would enhance the expression of HCP1. Furthermore, it is reported that cells surviving radiation acquire HIF-1 activity [31], and PDT could be an effective therapeutic procedure on radiation-resistant cells. Indeed, the amount of porphyrin that accumulated in the X-ray-resistant cells was greater than that in the control cells, and a significant increase in cytotoxicity with laser irradiation was observed in the X-ray-resistant cells (Figure 4 and Figure 5).

Although we have discussed the mechanism of porphyrin uptake into cells, the excretion mechanism is closely related to the amount of intracellular porphyrin. The extracellular efflux mechanism of porphyrins is associated with the ATP-binding cassette transporter G2 (ABCG2). We previously reported that the expression of the porphyrin efflux transporter ABCG2 decreases with an increase in intracellular ROS production, and that ABCG2 expression is elevated by treatment with antioxidants [32]. Continuous X-ray irradiation of RGK1 cells also increased intracellular ROS production, followed by HCP1 upregulation. Therefore, the porphyrin efflux transporter ABCG2 may be downregulated and porphyrin excretion may be attenuated. HCP1 upregulation and ABCG2 downregulation, induced by ROS production, elevated intracellular porphyrin accumulation and elicited an increase in cytotoxicity caused by laser irradiation in X-ray-resistant cancer cells.

Meanwhile, nitric oxide (NO) produced in cells may also be related to the enhancement of the PDT effect. High-dose X-ray irradiation of the mouse skin induces the expression of inducible NO synthase and increases NO production [33]. NO is also reported to be involved with HIF-1α stabilization and possibly associated with the elevation of HCP1 expression [26,34]. In addition, NO inactivates the enzyme ferrochelatase, which inserts ferrous iron into the porphyrin structure to produce heme, increasing the amount of porphyrin in the cells [35]. Therefore, the PDT-sensitizing effects may have been mediated by NO production in radiation-resistant cells. In addition to NO, another ROS, peroxynitrite, may be related to the expression of HCP1 and subsequent intracellular porphyrin accumulation. As mentioned, NO reacts highly with mitochondrial superoxide to produce peroxynitrite, a highly reactive oxygen species. Peroxynitrite also enhanced the expression of HCP1 and accumulation of porphyrin in cells [36]. Therefore, not only NO but also peroxynitrite, which is a reaction product of NO and superoxide, maybe overexpressed in X-ray-resistant cells and could be related to the cytotoxic effects of laser irradiation. In fact, the fluorescence intensity of HPF, which increases the fluorescence by reacting with highly reactive oxygen and nitrogen species such as hydroxyl radical and peroxynitrite, was elevated in the X-ray-resistant cancer cells compared to the control non-irradiated cells (Figure 2A,B).

## 5. Conclusions

The gastric cancer cell line RGK1 acquired X-ray resistance by continuous X-ray irradiation, and the established X-ray-resistant RGK1 cells overgenerated ROS from the mitochondria compared to the original RGK1 cells. We suggest that the excess amounts of mitochondrial ROS contribute to stabilizing HIF-1α and the subsequent induction of HCP1 expression, leading to the enhancement of porphyrin accumulation in the cells and the increase in cytotoxicity caused by laser irradiation. These results can benefit the clinical treatment of radiation-resistant cancer cells and improve their prognosis. However, this study was performed in vitro and its effect on animals remains unclear. We are currently investigating these effects in detail in animal models.

## Data Availability

All data are contained in the article.

## Supplementary Materials

The following supporting information can be downloaded at: https://www.mdpi.com/article/10.3390/pharmaceutics15112536/s1, Figure S1: The uncropped blots.

## References

[1] Lalo R., Zekja I., Kamberi F. (2023). Association of Cardiovascular Disease Risk and Health-Related Behaviors in Stroke Patients. Int. J. Environ. Res. Public Health.

[2] Toyoda K., Yasaka M., Iwade K., Nagata K., Koretsune Y., Sakamoto T., Uchiyama S., Gotoh J., Nagao T., Yamamoto M. (2008). Dual antithrombotic therapy increases severe bleeding events in patients with stroke and cardiovascular disease: A prospective, multicenter, observational study. Stroke.

[3] Alam M., Goldberg L.H. (2002). Serious Adverse Vascular Events Associated With Perioperative Interruption of Antiplatelet and Anticoagulant Therapy. Dermatolog. Surg..

[4] Louison S., Gabrielle P.H., Soudry A., Meillon C., Blanc J., Béal G., Arsène S., Le Mer Y., Berrod J.P., Kodjikian L. (2020). Perioperative risk of bleeding with antithrombotic agents in macular surgery: A national, prospective, multicentre study. Acta Ophthalmol..

[5] Vargason A.M., Anselmo A.C., Mitragotri S. (2021). The evolution of commercial drug delivery technologies. Nat. Biomed. Eng..

[6] Senapati S., Mahanta A.K., Kumar S., Maiti P. (2018). Controlled drug delivery vehicles for cancer treatment and their performance. Signal Transduct. Target. Ther..

[7] Ryan J.L. (2012). Ionizing Radiation: The Good, the Bad, and the Ugly. J. Investig. Dermatol..

[8] Naidu M.U.R., Ramana G.V., Rani P.U., Mohan I.K., Suman A., Roy P. (2004). Chemotherapy-induced and/or radiation therapy-induced oral mucositis—Complicating the treatment of cancer. Neoplasia.

[9] Langendijk J.A., Lambin P., De Ruysscher D., Widder J., Bos M., Verheij M. (2013). Selection of patients for radiotherapy with protons aiming at reduction of side effects: The model-based approach. Radiother. Oncol..

[10] Schlaff C.D., Krauze A., Belard A., O’Connell J.J., Camphausen K.A. (2014). Bringing the heavy: Carbon ion therapy in the radiobiological and clinical context. Radiother. Oncol..

[11] Arruebo M., Vilaboa N., Sáez-Gutierrez B., Lambea J., Tres A., Valladares M., González-Fernández Á. (2011). Assessment of the evolution of cancer treatment therapies. Cancers.

[12] Ohno T. (2013). Particle radiotherapy with carbon ion beams. EPMA J..

[13] Kamada T., Tsujii H., Blakely E.A., Debus J., De Neve W., Durante M., Jäkel O., Mayer R., Orecchia R., Pötter R. (2015). Carbon ion radiotherapy in Japan: An assessment of 20 years of clinical experience. Lancet Oncol..

[14] Galeaz C., Totis C., Bisio A. (2021). Radiation Resistance: A Matter of Transcription Factors. Front. Oncol..

[15] Alamilla-Presuel J.C., Burgos-Molina A.M., González-Vidal A., Sendra-Portero F., Ruiz-Gómez M.J. (2022). Factors and molecular mechanisms of radiation resistance in cancer cells. Int. J. Radiat. Biol..

[16] Nishida K., Tojo T., Kondo T., Yuasa M. (2021). Evaluation of the correlation between porphyrin accumulation in cancer cells and functional positions for application as a drug carrier. Sci. Rep..

[17] Castano A.P., Mroz P., Hamblin M.R. (2006). Photodynamic therapy and anti-tumour immunity. Nat. Rev. Cancer.

[18] Kaneko S., Kaneko S. (2016). Fluorescence-Guided Resection of Malignant Glioma with 5-ALA. Int. J. Biomed. Imaging.

[19] Hiyama K., Matsui H., Tamura M., Shimokawa O., Hiyama M., Kaneko T., Nagano Y., Hyodo I., Tanaka J., Miwa Y. (2013). Cancer cells uptake porphyrins via heme carrier protein 1. J. Porphyr. Phthalocyanines.

[20] Ito H., Matsui H., Tamura M., Majima H.J., Indo H.P., Hyodo I. (2014). Mitochondrial reactive oxygen species accelerate the expression of heme carrier protein 1 and enhance photodynamic cancer therapy effect. J. Clin. Biochem. Nutr..

[21] Ogura A., Oowada S., Kon Y., Hirayama A., Yasui H., Meike S., Kobayashi S., Kuwabara M., Inanami O. (2009). Redox regulation in radiation-induced cytochrome c release from mitochondria of human lung carcinoma A549 cells. Cancer Lett..

[22] Shimokawa O., Matsui H., Nagano Y., Kaneko T., Shibahara T., Nakahara A., Hyodo I., Yanaka A., Majima H.J., Nakamura Y. (2008). Neoplastic transformation and induction of H+, K+-adenosine triphosphatase by N-methyl-N′-nitro-N-nitrosoguanidine in the gastric epithelial RGM-1 cell line. Vitr. Cell. Dev. Biol.-Anim..

[23] Setsukinai K., Urano Y., Kakinuma K., Majima H.J., Nagano T. (2003). Development of novel fluorescence probes that can reliably detect reactive oxygen species and distinguish specific species. J. Biol. Chem..

[24] Li J.-M., Shah A.M. (2004). Endothelial cell superoxide generation: Regulation and relevance for cardiovascular pathophysiology. Am. J. Physiol. Integr. Comp. Physiol..

[25] Indo H.P., Yen H.C., Nakanishi I., Matsumoto K.I., Tamura M., Nagano Y., Matsui H., Gusev O., Cornette R., Okuda T. (2015). A mitochondrial superoxide theory for oxidative stress diseases and aging. J. Clin. Biochem. Nutr..

[26] Kurokawa H., Ito H., Terasaki M., Matano D., Taninaka A., Shigekawa H., Matsui H. (2019). Nitric oxide regulates the expression of heme carrier protein-1 via hypoxia inducible factor-1α stabilization. PLoS ONE.

[27] Koike M., Yutoku Y., Koike A. (2021). Inhibition of crandell-rees feline kidney cell proliferation by x-ray-induced senescence. J. Vet. Med. Sci..

[28] Majima H.J., Oberley T.D., Furukawa K., Mattson M.P., Yen H.C., Szweda L.I., St. Clair D.K. (1998). Prevention of mitochondrial injury by manganese superoxide dismutase reveals a primary mechanism for alkaline-induced cell death. J. Biol. Chem..

[29] Shayeghi M., Latunde-Dada G.O., Oakhill J.S., Laftah A.H., Takeuchi K., Halliday N., Khan Y., Warley A., McCann F.E., Hider R.C. (2005). Identification of an intestinal heme transporter. Cell.

[30] Klimova T., Chandel N.S. (2008). Mitochondrial complex III regulates hypoxic activation of HIF. Cell Death Differ..

[31] Harada H., Inoue M., Itasaka S., Hirota K., Morinibu A., Shinomiya K., Zeng L., Ou G., Zhu Y., Yoshimura M. (2012). Cancer cells that survive radiation therapy acquire HIF-1 activity and translocate towards tumour blood vessels. Nat. Commun..

[32] Kurokawa H., Ito H., Terasaki M., Matsui H. (2019). Hyperthermia enhances photodynamic therapy by regulation of HCP1 and ABCG2 expressions via high level ROS generation. Sci. Rep..

[33] Chi C., Ozawa T., Anzai K. (2006). In vivo nitric oxide production and iNOs expression in X-ray irradiated mouse skin. Biol. Pharm. Bull..

[34] Dewhirst M.W., Cao Y., Moeller B. (2008). Cycling hypoxia and free radicals regulate angiogenesis and radiotherapy response. Nat. Rev. Cancer.

[35] Yamamoto F., Ohgari Y., Yamaki N., Kitajima S., Shimokawa O., Matsui H., Taketani S. (2007). The role of nitric oxide in δ-aminolevulinic acid (ALA)-induced photosensitivity of cancerous cells. Biochem. Biophys. Res. Commun..

[36] Ito H. (2021). Peroxynitrite Production Induced by LPS and X-ray Treatment Enhances Cellular Incorporation of Porphyrin in Mouse RAW264 Macrophages. Appl. Sci..

