# Supplementary material for: Photodynamic Therapy for X-ray-Induced Radiation-Resistant Cancer Cells
Source: Pharmaceutics. 2023 Oct 26;15(11):2536. doi: 10.3390/pharmaceutics15112536 (PMC10674178; doi:10.3390/pharmaceutics15112536)
Supplement: Supplementary file 1 [file pharmaceutics-15-02536-s001.zip › pharmaceutics-2645939-supplementary.pdf]

Supplementary Materials

A

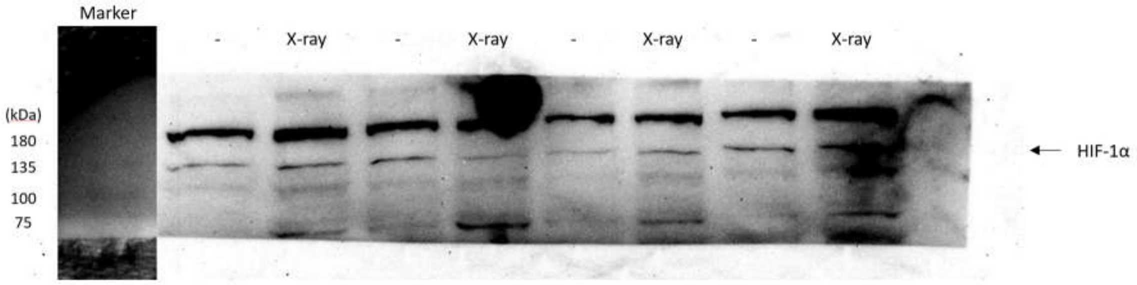

B

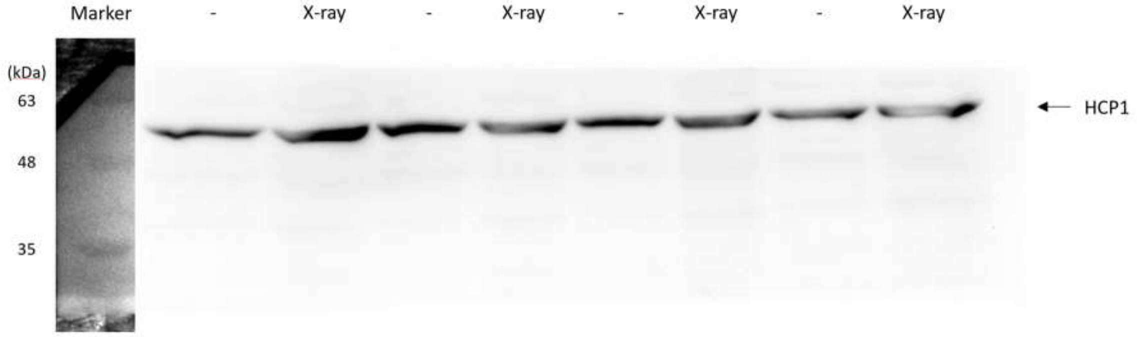

C

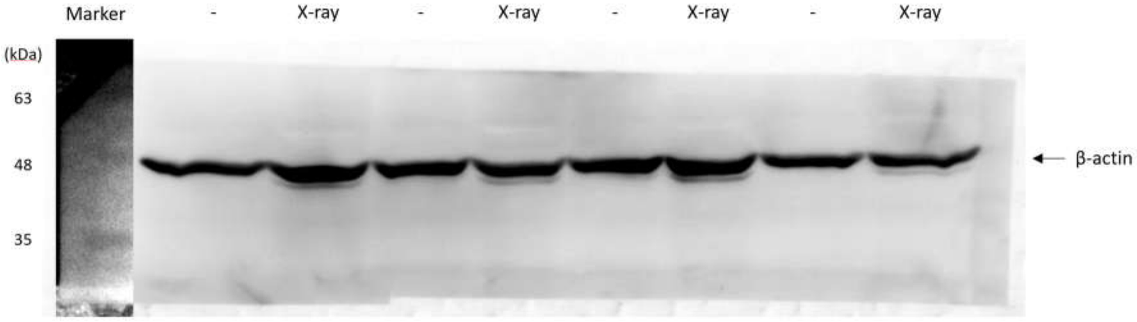

Figure S1. Uncropped Western blot figures of (**A**) HIF-1α, (**B**) HCP1, and (**C**) β-actin.
